# A Comprehensive Review of Treatment Approaches for Cutaneous and Genital Warts

**DOI:** 10.7759/cureus.47685

**Published:** 2023-10-25

**Authors:** Vaibhav B Kore, Ashish Anjankar

**Affiliations:** 1 Dermatology, Jawaharlal Nehru Medical College, Datta Meghe Institute Higher Education and Research, Wardha, IND; 2 Biochemistry, Jawaharlal Nehru Medical College, Datta Meghe Institute Higher Education and Research, Wardha, IND

**Keywords:** vaccination, cervarix, cryotherapy, warts, papilloma

## Abstract

Cutaneous and genital warts are common in both developed as well as developing countries. Human papillomavirus (HPV), which is a double-stranded DNA virus, is the causative agent of wart infection. Different types of HPV viruses are responsible for the different severity of diseases. Some types are associated with malignancy of the anal region and cervix. HPV is a common sexually transmitted disease in the United States. The incidence is most common in the younger age groups and the elderly population. Our main goal is to describe the different treatment modalities available for warts. Treatment modalities are divided into primary, secondary, and tertiary options. Topical medications, and physical excision of warts via cryotherapy, electrocautery, lasers, or photodynamic therapy are all common forms of treatment. Various clinical trials and randomized control trials have been seen as effective treatment against HPV infection. Higher remission rates are seen irrespective of different treatment options. Warts can be treated but the HPV virus cannot be completely removed. Older age, immunocompromised state, diabetes mellitus, and HIV are the predisposing factors for the disease. There is currently a large variety of medicines in use, all of which can differ significantly in terms of price, side-effect profiles, dosing regimens, length of therapy, and overall effectiveness. The best course of treatment has not yet been identified, and patients are often treated according to their unique needs.

## Introduction and background

Infection with the human papillomavirus (HPV), which is thought to be the cause of one of the most prevalent sexually transmitted diseases (STDs) in the United States, results in cutaneous and genital warts, also called condylomata acuminata. There are at least 80 identified HPV strains, and about 30 of these are known to lead to genital infections [1]. However, HPV types 6 and 11, sometimes referred to as the "low-risk" varieties since it is thought that they do not lead to malignant cellular alterations, are known to be the main causes of condylomata [2].

Genital warts are frequently noticed visually, negating the need for an additional biopsy. These exophytic lesions are characterized by hyperplastic squamous epithelium that displays koilocytes, which are squamous epithelial cells with an acentric, hyperchromatic nucleus displacement by a large perinuclear vacuole [3]. They develop as a result of the growth of the dermal papillae. Regarding differential diagnoses, syringomas and lichen nitidus should be considered in cases of plane warts on the face, and bowenoid papulosis and condylomata lata should be considered in cases of large verrucous lesions, such as those on the foot. Treatment should be started as soon as possible because each infection has a unique history and it is impossible to predict how warts will appear and spread. Given that treatment can stop the illness from becoming chronic or widespread in at least 50% of the affected patients, this could be regarded as a preventive measure [4].

The success of the treatment depends on several clinical parameters, including the severity of the disease, the size, and the location of the warts. This review describes the many approaches to preventing and managing cutaneous and genital warts. The HPV types in condylomata acuminata are depicted in Table 1 [5].

**Table 1:** Human papillomavirus types in condylomata acuminata

Low Risk	Intermediate Risk	High Risk
6, 11, 42, 43, 44, 53, 57, 81, 84	31, 33, 35, 45, 51, 52, 55, 56, 58, 59, 68	16, 18

## Review

Search methodology

**Figure 1:**
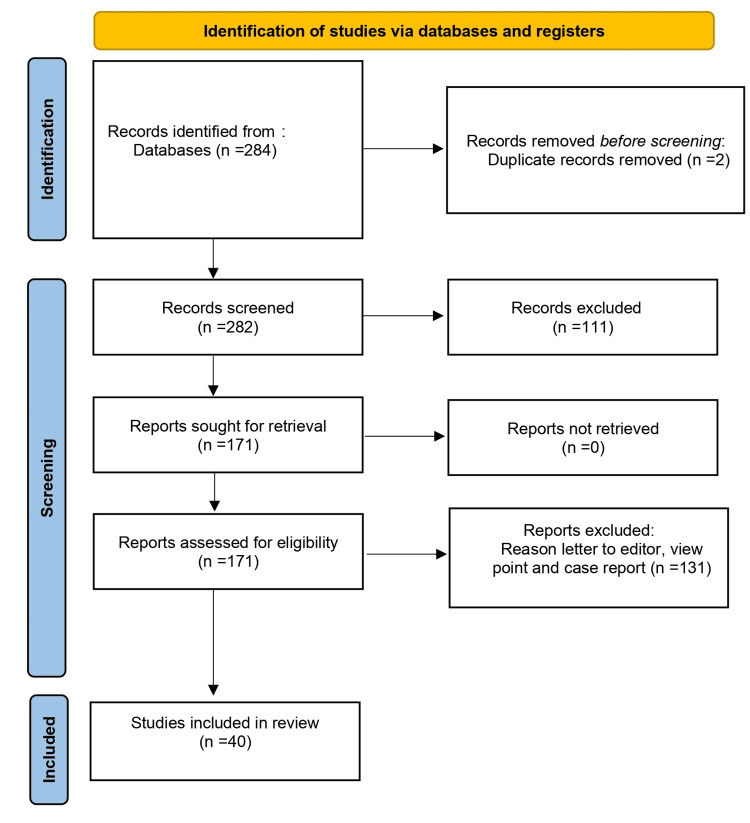
PRISMA flow diagram PRISMA: Preferred Reporting Items for Systematic Reviews and Meta-Analyses

To compile a comprehensive review of treatment approaches for cutaneous and genital warts, a systematic research methodology was adopted. Primary databases like PubMed, Google Scholar, and Web of Sciences were searched using keywords such as Cutaneous Warts, First Line Management, Second Line Management, Third Line Management, Genital Warts, Therapies, Vaccination, and Preventions, targeting publications in 2000-2023 in the English language. Emphasis was placed on peer-reviewed articles, clinical trials, and meta-analyses. Inclusion criteria focused on studies that focussed on the etiology and recent advances in cutaneous and genital warts. Exclusion comprised studies outside the set timeframe, non-peer-reviewed publications, and articles with a sole focus on recreational use. Once relevant articles were identified, data such as author names, publication year, study design, findings, and causes as well as modern treatment were meticulously extracted. Any overlapping or duplicate studies were filtered out to maintain the integrity of the review. To ensure organized citation and easy reference access, all sources were managed using the Zotero reference management software (Corporation for Digital Scholarship, Vienna, Virginia, United States). The Preferred Reporting Items for Systematic Reviews and Meta-Analyses (PRISMA) statement was followed for conducting this systematic review (Figure 1).

Cutaneous warts

The keratinocyte cell is damaged by the HPV, which has a stronger affinity for squamous epithelial tissue and causes papules or plaque on the skin and has a rough surface. Before beginning treatment, risk factors should be identified and treated accordingly. 

First-Line Management

Salicylic acid:This is a simple and cost-beneficial treatment of choice for cutaneous warts. Salicylic acid is thought to trigger the local immune response even though its primary effects on HPV-induced hyperkeratosis are chemical ablation and irritation. Salicylic acid can be purchased without a prescription and comes in a variety of forms and dosages. Salicylic acid 17% is most frequently used; however, there isn't enough information to say that one preparation or concentration is better than another [6]. The following therapeutic protocols are employed. A hot shower or bath is taken to get rid of hyperkeratotic lesions. the treatment plan suggests daily application for 7-12 weeks. Application on face and eyes should be avoided [7].

Cryotherapy: For the treatment of warts, cryotherapy is frequently used in hospitals and is also accessible over the counter. Primary care physicians typically utilize liquid nitrogen, which can freeze tissue to a temperature of 321°F (196°C), and over-the-counter cryotherapy devices can only freeze tissue to a temperature of 94°F (70°C). Liquid nitrogen can be applied using a piece of cotton swab or a cryogen. Cryotherapy and salicylic acid combination therapies had greater rates of elimination than monotherapies [8]. There should be at least three months of therapy. Pain, blistering, hypo- or hyperpigmentation, especially in dark skin, tendon or nerve injury with vigorous therapy, and onychodystrophy after treatment of periungual warts are common side effects of cryotherapy [9].

*Second-Line Management* 

CO_2_ laser and electrocautery: Common wart or cutaneous wart removal is categorized under the category of ablative operations (physical annihilation). For the treatment of warts, CO_2_ laser is commonly employed at medical offices and is also accessible over the counter. Doctors working in primary care typically utilize liquid nitrogen, which can freeze tissue to a temperature of 321°F (196°C) as over-the-counter CO_2_ laser devices can only freeze tissue to a temperature of 94°F (70°C). Using a cotton pad or a cryogen, liquid nitrogen can be applied [10]. When treating a plantar wart, the wart's skin should be scraped using a blade. Next, nitrogen should be administered until a 2 mm white halo is evident, and repeated if necessary [11]. Scarring is a possibility with both CO_^2^_ lasers and electrocautery techniques. Argon plasma coagulation may also be considered as an ablative technique if the necessary tools are available, particularly for condyloma acuminata [12].

Photodynamic therapy: The cornerstone of photodynamic therapy is treatment with aminolevulinic acid (LEVULAN® KERASTICK®), which is followed by phototoxicity. A photosensitizer known as aminolevulinic acid works by causing the oxidation process in aberrant cells after exposure to visible light [13]. The bump is often treated with amino-levulinic acid for three to eight hours, following which the region is subjected to various light sources [14]. Actinic keratosis, basal cell carcinoma, intraepithelial lesions of the vulva and anus, as well as genital warts, are just a few of the skin conditions that can be treated with photodynamic treatment. All warts in this therapy are given keratolytic treatment before phototherapy, and recent recommendations link sufficient paring with better phototherapy outcomes [15]. Some people may have negative side effects including burning, itching, and excruciating discomfort. Despite the likelihood that photodynamic therapy may be helpful, it is costly and less accessible to medical providers compared to different treatments and typically performed by dermatologists.

Third-Line Management

Imiquimod cream:Imiquimod is the most commonly used chemotherapeutic agent. Adults with cutaneous warts can now be treated with imiquimod 5% cream. Interferon-alpha and other cytokines are released as a result, which prevents virus replication [16]. Various cytokines, including interferon, interleukin-6, and tumor necrosis factor, are released as a result of immune system activation and are essential for triggering an inflammatory response that promotes wart removal [17,18]. Infusions of the *Candida* fungal antigen as an immunomodulator intravenously for persistent, non-genital warts are helpful. Mumps antigen may be tried for treatment. Fever and arthralgia are examples of side effects [19]. Levamisole is an immunomodulator used in the treatment of various viral etiology such as genital wart and molluscum viruses. Levamisole stimulates the breakdown of the cyclic amp which results in a chemotactic response.

Bleomycin, 5-fluorouracil (5-FU), and cidofovir injections: Chemotherapeutic bleomycin prevents cells and viruses from synthesizing DNA. It results in acute tissue necrosis, which could trigger an immunological reaction [20]. Intralesional injection of 5-FU can be used to treat cutaneous warts, but it often produces pain and ulceration and it is not widely used. Cidofovir is an antiviral analog that is used in the treatment of cytomegalovirus-induced retinitis; it is also effective against the treatment of HPV, Epstein-Barr virus, and other DNA viruses [21].

Genital warts

Once an individual has contacted HPV, the virus normally needs approximately 10-14 weeks of incubation time before showing any clinical symptoms. Physical symptoms typically start 8-10 weeks after the first contact with the virus [22]. However, the virus can remain dormant inside epithelial cells for a lengthy time. As a result, an individual may have an infection for their entire lifespan without experiencing any clinically documented warts. As evidenced by the discovery of positive viral samples during DNA analysis of allegedly uninfected vaginal tissue, many studies place the prevalence of latent HPV infection as high as 40% [23]. Genital warts may grow larger and more numerous after the first clinical presentation or, conversely, they may spontaneously shrink [5]. In fact, following the early four-month period of infection, almost 30% of all warts will decline. Even after receiving the necessary treatments, most of these genital warts will return three months following infection [24]. Until recently, podophyllotoxin, cryotherapy, and laser treatment were the only treatments available for condyloma acuminata. The range of therapeutic choices has widened with new pharmacological substances and techniques. The current evaluation aims to give a review of all currently available genital wart therapies and to assess the value of innovative treatments.

Topical Agents

Podophyllotoxin: An isolated podophyllum plant extract called podophyllotoxin adheres to intracellular tiny tubes. slows mitotic division, and causes wart necrosis that peaks three to five days after injection. As the lesions necrotize and recover in a short period, shallow erosions happen [25]. This course of treatment is typically regarded as safe, efficient, and self-administrable. Podophyllotoxin is in a gel form that should be applied two to three times daily for a minimum of three days, of the week. Treatment is for four to six weeks. For penile lesions, a solution is typically advised, whilst cream or gel preparations are regarded to be more palatable for use on anal or vaginal lesions [26]. Podophyllotoxin not only causes local reactions (erythema, edema, erosions), but it can also have a negative impact on the liver, induce hepatic dysfunction, have neurological side effects, produce hallucinations, cause psychoses, cause epistaxis, and other symptoms [27].

Imiquimod and diphenylcyclopropenone:Imiquimod is an immunity modulator that works by directly activating innate immune cells via the toll-like receptor 7 (TLR-7). After interacting with TLR7, imiquimod causes dendritic cells to produce a potent pro-inflammatory response in vitro. Additionally, imiquimod topical application causes the functional development of epithelial Langerhans cells in vivo, boosts these cells' migration to local lymph nodes, and supports a Th1-biased and specific antigen CD8+ T cell response [28]. Localized erosions, erythema, and burning are some of the most typical side effects.

Trichloroacetic acid (TCA): The skin and mucosa are damaged chemically by TCA, which cauterizes, burns, and erodes them. TCA often requires a doctor to administer it because it is manufactured in 80-90% solutions. Warts can occasionally be successfully treated with just one dosage, but more often than not, many treatments are needed [29]. TCA is a cheap and effective medication, although it does need regular use and adherence to a regimen. The product's destructive nature frequently causes the underlying viral infection as well as the superficial wart to spread, leading to clearance rates that have been estimated to be 70-80% and high recurrence rates of 36% [30]. In Taner et al.'s study, despite the frequent occurrence of transitory searing discomfort during its application, none of the patient groups stopped their medication [30].

Electronics-Based Therapy

Cryotherapy: The most common method of ablative treatment approach is cryotherapy, which is similar to the management of extragenital warts. The largest possibility of clearance rate is induced by cryotherapy, which is suitable for the treatment of multiple wart lesions [31]. Through the procedure of cryotherapy, the aberrant tissue is rendered immobile by the application of a chilling substance, such as liquid nitrogen or nitrous oxide. Extremely low temperatures are required to permanently harm vascular and cutaneous tissue. This triggers an immunological repair reaction, which causes the necrosis and removal of the damaged cells. This treatment is generally beneficial for numerous warts. There is less likelihood of recurrence after using cryotherapy ablation. Cryotherapy has drawbacks such as the need for several outpatient sessions and the pain that can prevent some patients from receiving it repeatedly. However, because cryotherapy only has local effects, it is currently the treatment of choice for expecting mothers who have several warts [32].

CO_2_ laser therapy: A focused beam of infrared light energy is used in CO_2_ laser therapy to heat and eventually vaporize the areas that are being treated. The additional advantage of the high light intensity is that it immediately cauterizes any ligated vessels, guaranteeing a nearly bloodless treatment. The laser beam's spatial confinement enables precise tissue ablation, resulting in quick recovery with minimal to no scarring. Sadly, laser therapy is a treatment option that is both expensive and challenging. Physicians themselves must need further training to efficiently use the specialized laser equipment, which must be acquired and maintained continuously. Additionally, the vaporization of viral lesions may cause the release of HPV DNA into the environment [33]. It is necessary to take the proper precautions to ensure that medical professionals and support staff are immune from illness. Due to this, special, virus-resistant masks and a vacuum ventilation system are required in the exam room.

Newer Therapy

Interferon therapy, podophyllotoxin, and 5-FU are not advised for routine usage in the primary care context due to their limited efficacy and toxicity. The first local therapy for genital warts was podophyllotoxin, although samples of the medicine differed widely in terms of its active ingredient due to a lack of standardized drug manufacturing. Negative skin effects like burning, redness, discomfort, itching, or swelling became more likely as a result. Extremely rare, enteritis, bone marrow suppression, gastrointestinal pain, and neurological deterioration have all been connected to overusing podophyllotoxin and high systemic absorption [34]. When administered alone, podophyllotoxin is typically regarded as being less successful than podophyllotoxin, cryotherapy, or electrosurgery in the treatment of genital warts. One of the first chemotherapeutics, 5-FU has been used successfully to treat cancer for a period of over 40 years. Local 5-FU continues to be recognized as a promising treatment for urethral warts, despite not having FDA approval for use in the management of genital warts [35]. Aside from having slightly more severe side effects than imiquimod 5% cream with equivalent clearance rates, the application of 5-FU has previously been linked to highly varied response rates.

Vaccination and Prevention

The HPV vaccine is recommended to be administered in the early adolescent years since it is most efficient before sexual activity-related HPV exposure. Obstetricians-gynecologists and other physicians could participate in shared clinical decision-making regarding HPV vaccination for some women aged 27-45 who had never received the vaccine, taking into account the patient's risk of contracting a new virus associated with HPV and the possible benefits of the HPV vaccine. Women aged 27-45 who are more likely to be exposed to or acquire HPV, including younger women, those who are not in established committed partnerships, and those who have recently been diagnosed with STDs would get the most advantages from vaccination. It is not typically advised for women in long-term monogamous relationships [36].

Gardasil

It is the first HPV vaccine that the FDA has approved. The recombinant, quadrivalent vaccine was created to prevent the diseases caused by HPV types 6, 11, 16, and 18: cervical, vulvar, and vaginal cancer, as well as condyloma acuminata in girls and young women aged 9-26 [37]. Additionally, Gardasil is recommended for the prevention of lesions that are precancerous or dysplastic, and are brought on by HPV 6, 11, 16, and 18. The HPV subtypes, that have directly responsible for roughly 90% of genital warts and 70% of cervical malignancies, are triggered by Gardasil to produce host antibodies. In patients who never had HPV infection, three different doses of the injectable Gardasil appear to be 99% successful in preventing the development of genital warts [38]. Fainting, inflammation at the injection site, headaches, nausea, and fever are the most common moderate HPV vaccination adverse effects.

Cervarix

It protects against HPV 16 and 18 and is a recombinant bivalent vaccination. HPV 16 and 18 are two carcinogenic kinds that are targeted by Cervarix and are linked to cancer of the cervical region, cervical intraepithelial neoplasia grade 1 or even worse, and in situ malignancy. Similar to the HPV 4 safety information, there were significantly greater reports of discomfort, redness, and swelling at the injection site in the HPV 2 group than in the placebo group. The most prevalent general complaints were fatigue, headaches, and myalgia [39]. Modifications in preparation, specifically in relation to adjuvant components that boost the body's immune response to vaccination antigens, may be the cause of the observed variations in immunological response elicited by the two vaccines. They may serve as predictors of the length of protection from HPV 16 and HPV 18 even though the therapeutic significance of this immune response variation is unknown [40].

## Conclusions

Among the most prevalent STDs infecting people of all ages are external genital warts and the HPV infections associated with them. To combat the underlying viral infection, current treatment approaches only target exterior warts and have thus shown to be ineffective in producing lasting effects. Topical, surgical, and immunomodulatory treatments fall into several categories and have varying costs, lengths of treatment, dose regimens, and side effects. One particular technique has not yet proven itself as the gold standard for treatment, and there is currently little data to support the idea that a particular class of therapy is not more successful than others. The demands and preferences of each patient are normally taken into account while choosing a therapy approach. Considering people's startlingly high genital wart incidence and a shortage of effective treatments, HPV vaccinations like Gardasil and Cervarix may significantly lower the impact of infectious diseases by avoiding viral spread and infection.
